# Hyperoxia-Induced miR-195 Causes Bronchopulmonary Dysplasia in Neonatal Mice

**DOI:** 10.3390/biomedicines12061208

**Published:** 2024-05-29

**Authors:** Patrick Philpot, Fred Graumuller, Nicole Melchiorre, Varsha Prahaladan, Xander Takada, Srinarmadha Chandran, Melissa Guillermo, David Dickler, Zubair H. Aghai, Pragnya Das, Vineet Bhandari

**Affiliations:** 1 Division of Neonatology, Department of Pediatrics, Thomas Jefferson University, Nemours, Philadelphia, PA 19107, USA; pat.philpot@gmail.com (P.P.); zubair.aghai@nemours.org (Z.H.A.); 2 Division of Neonatology, Department of Pediatrics, Drexel University College of Medicine, Philadelphia, PA 19102, USA; varsha0mp@gmail.com (V.P.); das-pragnya@cooperhealth.edu (P.D.); 3 Division of Neonatology, The Children’s Regional Hospital at Cooper, Cooper Medical School of Rowan University, Camden, NJ 08103, USA; graumuller-fred@cooperhealth.edu (F.G.); melchi23@rowan.edu (N.M.); takada-xander@cooperhealth.edu (X.T.); srinarmadhachandran@gmail.com (S.C.); mtguillermo@hotmail.com (M.G.); dickle45@rowan.edu (D.D.)

**Keywords:** lung injury, preterm newborn, chronic lung disease, premature infant, microRNA, targeted therapy

## Abstract

**Background:** Exposure to hyperoxia is an important factor in the development of bronchopulmonary dysplasia (BPD) in preterm newborns. MicroRNAs (miRs) have been implicated in the pathogenesis of BPD and provide a potential therapeutic target. **Methods:** This study was conducted utilizing a postnatal animal model of experimental hyperoxia-induced murine BPD to investigate the expression and function of miR-195 as well as its molecular signaling targets within developing mouse lung tissue. **Results:** miR-195 expression levels increased in response to hyperoxia in male and female lungs, with the most significant elevation occurring in 40% O_2_ (mild) and 60% O_2_ (moderate) BPD. The inhibition of miR-195 improved pulmonary morphology in the hyperoxia-induced BPD model in male and female mice with females showing more resistance to injury and better recovery of alveolar chord length, septal thickness, and radial alveolar count. Additionally, we reveal miR-195-dependent signaling pathways involved in BPD and identify PH domain leucine-rich repeat protein phosphatase 2 (PHLPP2) as a novel specific target protein of miR-195. **Conclusions:** Our data demonstrate that high levels of miR-195 in neonatal lungs cause the exacerbation of hyperoxia-induced experimental BPD while its inhibition results in amelioration. This finding suggests a therapeutic potential of miR-195 inhibition in preventing BPD.

## 1. Introduction

Bronchopulmonary dysplasia (BPD) is the most common chronic respiratory disease in infants, occurring in about 43% of infants born at or before 28 weeks of gestation and in those with very low birth weight (<1250 g) [1,2]. The incidence of BPD has increased, at least in part, because and despite medical advances in neonatal care which have increased the survival rate of extremely preterm infants [3]. BPD is characterized by alveolar simplification, impaired lung growth, dysregulated blood vessel development, and abnormal pulmonary function [4]. It is associated with substantial morbidity and mortality in premature infants and predisposes individuals to the development of lifelong, chronic respiratory diseases in adulthood, including asthma and chronic obstructive pulmonary disease (COPD), thereby presenting a devastating, sustained, and increasing burden for patients and healthcare providers alike [5]. Substantial sex-specific differences exist in the development and the severity of BPD, whereby male infants are more severely affected than their female counterparts [6,7].

MicroRNAs (miRs) are short (20–24 nucleotides) non-coding RNA molecules that act as negative regulators in target gene expression at the post-transcriptional level [8]. They regulate many biological processes and are necessary for maintaining cellular homeostasis. miRs constitute a necessity for regulating nearly all biomolecular processes which include metabolism, proliferation, migration, inflammation, apoptosis, and differentiation. Given their importance, the deregulation of miR expression is essential to the pathogenesis of many human diseases including almost all cancers. As such, miRs are promising future targets for prognosis, diagnosis, and therapy. Previous studies have linked anomalous miR expression patterns to the pathogenesis of numerous lung diseases such as COPD, lung cancer, asthma, and BPD [9,10]. However, the expression and function of miR-195 in BPD remains unknown.

Hence, we evaluated the expression and functional relevance of miR-195 within our experimental models of mild, moderate, and severe BPD [11]. In addition, we also focused on male–female differences, given their importance in both experimental and human BPD [6,7].

In this study, we demonstrate that miR-195 levels are significantly increased in male and female neonatal mice lungs with exposure to moderate hyperoxia (60% O_2_) when compared to room air (RA) controls. The inhibition of miR-195 by an antagomir minimized progression towards the BPD lung morphology after hyperoxia exposure. Furthermore, we identified PH domain leucine-rich repeat protein phosphatase 2 (PHLPP2) as a functional target of miR-195 in BPD and found it to be inhibited in hyperoxia-induced BPD. We also show that PHLPP2 suppression induced an inversely proportional upregulation of p-Akt. Female mice showed similar trends as those observed in male mice in response to hyperoxia; however, we noted some sex-specific differential responses in their target gene expression as well as in their morphology. Lastly, we report that the expression of miR-195 in human tracheal aspirates (TAs) was increased in infants who went on to develop BPD. Collectively our findings suggest a therapeutic potential for miR-195 as a novel target in regulating hyperoxia-induced BPD, particularly at levels of O_2_ exposure (mild–moderate BPD) that hold translational significance as they are currently being utilized in the treatment of infants with this disease in the neonatal intensive care unit (NICU).

## 2. Materials and Methods

### 2.1. Animals

All experimental wild-type (WT) mice of the C57BL/6 strain were purchased from the Jackson Laboratory (Bar Harbor, ME, USA) and housed in the Drexel University College of Medicine (Philadelphia, PA, USA) and Cooper University Hospital (Camden, NJ, USA) vivarium animal care facilities. The selection of the C57BL/6 strain was based on its reported sensitivity to hyperoxia-induced lung injury, as previously reported [12]. Mice were allowed free access to standard food and water. All animal experiments were conducted in accordance with NIH policies and were approved by the Institutional Animal Care and Use Committees (IACUC) at Drexel University College of Medicine and Cooper University Hospital. Both male and female newborn mice were used for the experiments, at pre-specified postnatal (PN) ages.

### 2.2. Hyperoxia Exposure

Newborn (NB) mice (along with their mothers) were exposed to hyperoxia by placing them in cages in a medium-sized airtight Plexiglas container (55 × 40 × 50 cm) (Bio-Spherix, Parish, NY, USA), following standard methods [8]. All NB mice were exposed to the 3 different oxygen concentrations in separate experiments. To ensure adequate nourishment for the mouse pups, the dam was replaced every other day with another lactating dam to prevent oxygen toxicity to the adult animal. In our experimental model of BPD, NB mice were exposed to different concentrations of O_2_ (as specified below) from PN day 1 to PN day 4, which corresponds to the saccular stage of lung development in rodents. Mice were then allowed to recover in RA for 10 days (i.e., during the alveolar stage of lung development). The lungs were harvested at PN day 14, as previously described [13]. Oxygen concentrations during the first 4 days of life were 40%, 60%, or 100% to induce mild, moderate, and severe BPD, respectively. Only one oxygen concentration was applied to one litter of mouse pups in one experiment (Supplemental Figure S1). The inside of the chamber was kept at atmospheric pressure, and mice were exposed to a 12 h light–dark cycle. Litter size was limited to six pups per dam to control for the effect of litter size on nutrition and growth. A cocktail of xylazine–ketamine was used for anesthesia prior to harvesting the NB mice lungs. The NB mice were sacrificed and the lungs were procured for RNA, protein, and histology analyses on PN day 14.

### 2.3. Cell Culture

Neonatal mouse Type 2 alveolar epithelial cells (TIIAECs) (ScienCell, Carlsbad, CA, USA) were grown in Dulbecco’s Modified Eagle’s Medium (DMEM), supplemented with 2% Fetal Bovine Serum (FBS), 1% penicillin–streptomycin solution, 1% L-glutamine, 0.1% insulin–transferrin–sodium selenite, and 0.1% epidermal growth factor (EGF), per the manufacturer’s instructions (Cell Biologics kit M6621, Chicago, IL, USA) at 37 °C in 95% air/5% CO_2_. Neonatal mouse microvascular lung endothelial cells (MLECs) were grown in DMEM, supplemented with 0.1% of each vascular endothelial growth factor (VEGF), heparin, endothelial cell growth supplement (ECGS), hydrocortisone, 1% L-glutamine, 1% penicillin–streptomycin solution, and 5% FBS (Cell Biologics kit M1168, Chicago, IL, USA). Hyperoxia conditions were implemented by placing the cells at 70–80% confluence for specified time points in 40% O_2_ at 37 °C in a tightly sealed modular chamber (Billups-Rothenburg, San Diego, CA, USA).

### 2.4. Western Blot

Western blotting was performed as previously described [8]. Briefly, whole-lung lysates and cell extracts were made in radioimmunoprecipitation assay (RIPA) buffer, and the protein concentration was determined using the bicinchoninic acid (BCA) assay (Thermofisher Dye, ThermoFisher Scientific, Waltham, MA, USA). Proteins were loaded at 30 µg per well and isolated using sodium dodecyl sulfate–polyacrylamide gel electrophoresis (SDS PAGE; at 4–20% concentration) before they were transferred onto nitrocellulose membranes (BioRad, Hercules, CA, USA), which were blocked for 1 h at room temperature in 3% bovine serum albumin (BSA) in tris-buffered saline with 0.1%Tween 20 (TBST) and incubation with primary antibodies overnight at 4 °C. The following day, membranes were washed three times with TBST, incubated with the fluorescent-conjugated secondary antibody for 1 h at RT, washed three times with TBST, and analyzed using LICOR infrared imaging. Equality of loading was confirmed by probing for vinculin. The primary antibodies used were PHLPP2 (ProteinTech, Rosemont, IL, USA; 25244-1-AP; 1:500), Akt (Cell Signaling Technology or CST, Danvers, MA, USA); 9272S; 1:1000), p-Akt (CST; 12694; 1:500), Vinculin (Santa Cruz Biotechnology, Dallas, TX, USA (SC; 7361; 1:1000)), Mitofusin 2 (SC; 515647, 1:500), interleukin (IL)-1β (CST; 12242, 1:500), p53 (CST); 2524; 1:1000), and Bcl-2 (CST; 2870; 1:1000).

### 2.5. Lung Bronchoalveolar Lavage Fluid (BALF), Histology, and Morphometry

Both the RA control and BPD mice were anesthetized using a cocktail of xylazine and ketamine. The trachea of each mouse was cannulated by endotracheally instilling Phosphate-buffered saline (PBS) to achieve lung inflation. To collect the bronchoalveolar lavage fluid (BALF), the mouse airways were washed two times using a 30-gauge syringe needle with a volume of 200 μL of 1% PBS and pooled. BALF was centrifuged at 4 °C at 1000 × *g* for 10 min and the supernatant was separated, collected, and later used to quantify total protein, as previously described [10]. Total protein was quantified using the Pierce BCA Protein Assay Kit (Fisher Scientific Co, Houston, TX, USA). To analyze changes in the morphology of the lung following hyperoxia exposure, tissues were first inflated via perfusion with 1% PBS and subsequently fixed via immersion overnight in 4% paraformaldehyde. The lung tissue was then washed in PBS, dehydrated using 70% ethanol, and embedded in paraffin. Lastly, 5 μm thick sections were cut from the paraffin blocks and were then stained with hematoxylin and eosin (H&E), as previously described [10].

Lung sections were imaged at 10× and 20× total magnification using an Olympus IX70 microscope with a DP73 camera and Olympus CellSens software (version 7; Olympus Life Science, Waltham, MA, USA) by choosing at least three areas at random. We excluded large airways and blood vessels when selecting the random areas. Chord length, septal thickness, and radial alveolar count were then measured using a customized macro of the Analyze Particles function of ImageJ version 1.5 (NIH, Bethesda, MD, USA; http://imagej.nih.gov/ij) (accessed on 21 July 2015) [13].

### 2.6. Quantitative Real-Time PCR 

RNA from homogenized lung lysates from RA and BPD mice was extracted using the TRIzol reagent (Invitrogen, Carlsbad, CA, USA). One µg of total RNA was utilized using the High-Capacity Reverse Transcription cDNA Kit (Applied Biosystems, Foster City, CA, USA) for regular cDNA synthesis and the miScript II RT Kit (Qiagen, Germantown, MD, USA) for miR-195 cDNA synthesis. For RNA quantification, real-time PCR in a 20 µL volume was carried out using the Power SYBR Green (Applied Biosystems, Foster City, CA, USA) for mRNA and the QuantiTect SYBR Green PCR Kit (Qiagen, Germantown, MD, USA) was used for miR-195 quantification. RNU6 was used as the reference gene for miRNA analysis for all lung samples.

### 2.7. miRNA Mimic and Inhibitor Experiments

Two doses of 20 µM in a volume of 3 µL/nostril of miR-195 mimic or inhibitor (Qiagen, Germantown, MD, USA) were administered intranasally to pups on PN2 and PN4, as previously described [8].

### 2.8. Human TA

TAs were collected from intubated infants born at less than 32 weeks of gestational age (GA), employing a routine suction technique within the first 14 days of life. Pertinent clinical information was obtained from the electronic medical record, including but not limited to GA at birth, post-menstrual age (PMA), ventilator settings, and the fraction of inspired oxygen (FiO_2_) at the time of collection. The TA pellet samples were transported on ice and stored at −80 °C until analysis. We used the NIH 2001 consensus definition for identifying infants with BPD as this definition has been validated in other studies and allowed for the identification of infants with established BPD at 36 weeks PMA [4]. The collection of human TAs from intubated neonates in the NICU was approved by the Institutional Review Board at Cooper and informed signed consent was obtained from one or both parents prior to the collection of samples. 

### 2.9. Statistical Analyses

Results are presented as the means ± standard error of the mean (SEM). Data sets are tested for normality using the Shapiro–Wilk normality test and compared using Student’s two-tailed unpaired *t*-test or 1-way ANOVA (followed by Tukey’s Multiple Comparison post hoc test) in GraphPad Prism 9.0 (GraphPad Software, Inc., San Diego, CA, USA). A *p* value of less than 0.05 was taken to be statistically significant. 

## 3. Results

**Figure 1:**
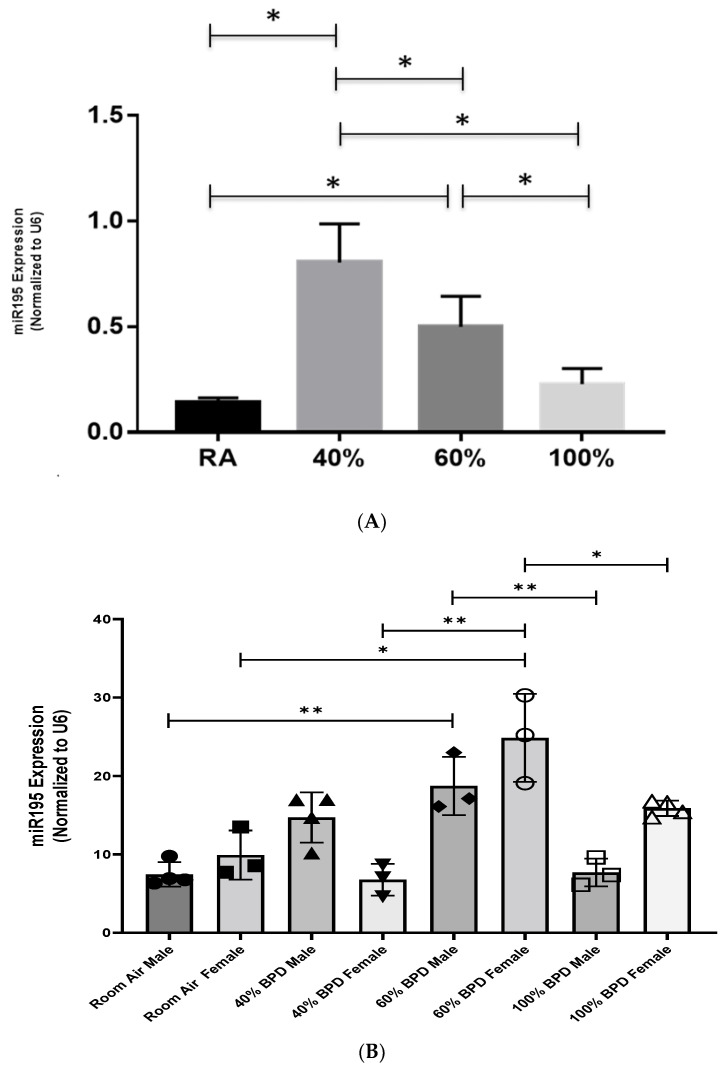
Expression of miR-195 in hyperoxia-exposed NB lungs. (**A**). NB mice were exposed to different concentrations of oxygen (40, 60, and 100%) from PN day 1 to 4 and recovered in room air until they were sacrificed on PN day 14. RA: room air. A minimum of four animals were used in each group (male and female combined). * *p* < 0.05. (**B**). Sex-dependent differences of miR-195 expression at varying O_2_ concentrations used for hyperoxia-induced experimental bronchopulmonary dysplasia (BPD). n = 3–4. * *p* < 0.05, ** *p* < 0.01.

**miR-195 expression in developing whole lungs and lung cells with exposure to varying concentrations of oxygen.** To assess the role of miR-195 in hyperoxia-induced lung disease in neonates, we exposed NB WT mice to varying degrees of hyperoxia: 40%, 60%, and 100% O_2_ from PN day 1 to 4. Thereafter, the mice were allowed to recover in RA until PN day 14. Next, we compared the expression patterns of miR-195, and a significant increase could be observed with exposures to 40% O_2_ (mild BPD model) and 60% O_2_ (moderate BPD model) hyperoxia, with expression levels decreasing to becoming comparable to that of the RA group in mice exposed to 100% O_2_ (severe BPD model) (Figure 1A).

Subsequently, we measured the expression levels of miR-195 in mouse neonatal TIIAECs and MLECs for 16 h. While TIAECs cover 90% of the alveolar surface and are very susceptible to oxygen toxicity, the TIIAECs are the surfactant-producing cells and the progenitor cells for the TIAECs. Hence, we studied the TIIAECs to evaluate their response to hyperoxia exposure as it would directly impact the surface tension-lowering function of the lung as well as provide an indirect measure of gas exchange function. We utilized 40% O_2_ exposure given the highest level of miR-195 expression in whole-lung lysates was demonstrated at this concentration. As shown in Supplemental Figure S2A,B, miR-195 expression increased at 4, 8, and 16 h in the TIIAECs, while no interpretable change could be observed in endothelial cells. 

Next, we wanted to assess if there were measurable differences in miR-195 expression between male and female mouse pups in whole-lung lysates.

**Sex differences in miR-195 expression in developing lungs upon exposure to varying concentrations of oxygen.** Given the known sex specificity in BPD [6], we then checked to see if there were any differences between males and females in the expression of miR-195 in whole-lung lysate samples. As shown in Figure 1B, no significant difference between males and females in miR-195 expression could be observed in RA. While there was a trend towards increased expression in males at 40% O_2_ (mild) BPD, miR-195 expression significantly increased at 60% O_2_ (moderate) BPD (Figure 1B). A similar increase was also found at 60% O_2_ (moderate) BPD in female mice (Figure 1B). 

Thus, taken together, these data suggest that miR-195 expression increased in response to hyperoxia in developing male lungs, with the most significant increase in mild–moderate, but not in severe BPD models. Female mice showed a similar trend as those observed in male mice in response to hyperoxia, with significantly increasing miR-195 expression levels in the 60% (moderate) O_2_ exposure BPD model. TIIAECs (but not endothelial cells) exposed to 40% O_2_ showed a significant increase in miR-195 in a similar manner as observed in whole-lung lysates. 

Next, we investigated the downstream intracellular signaling of miR-195 to further characterize its effects in NB mouse lung tissue within the context of BPD.

**miR-195 downregulates PHLPP2 in developing lungs upon exposure to varying concentrations of oxygen.** To identify downstream cell signaling circuitry, we focused on PHLPP2 as a known downstream target protein of miR-195 [14]. 

**Figure 2:**
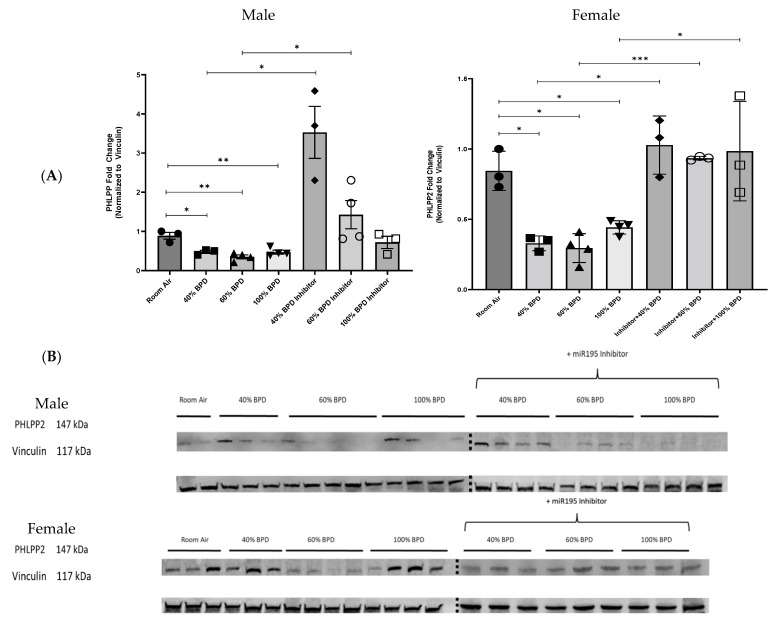
miR-195 inhibition increased PHLPP2 expression in the lungs of NB male and female mice in hyperoxia-induced experimental BPD. (**A**). Fold-change of relative protein expression. (**B**). Representative Western blot images showing increased PH domain leucine-rich repeat protein phosphatase 2 (PHLPP2) expression hyperoxia exposure with miR-195 inhibition. Each well represents lung tissue from an individual mouse. n = 3–4. * *p* < 0.05, ** *p* < 0.01, *** *p* < 0.001.

Upon using the miR-195 mimic in the 40% BPD model, we noted a marked decrease in PHLPP2 expression (Supplemental Figure S3A). To further evaluate the functional role of miR-195 in the development of experimental BPD, we examined PHLPP2 expression in the lungs of hyperoxia-induced BPD mice after the intranasal administration of the miR-195 inhibitor (antagomir). In male mice, the inhibition of miR-195 restored expression levels of PHLPP2 at 40% O_2_ (mild) BPD and 60% O_2_ (moderate) BPD (Figure 2A,B). In female mice, this restoration of PHLPP2 was more consistent across all three—mild, moderate, and severe—BPD models (Figure 2A,B). This finding suggested a direct causal relationship between miR-195 expression and PHLPP2 inhibition in both male and female BPD models.

**Figure 3:**
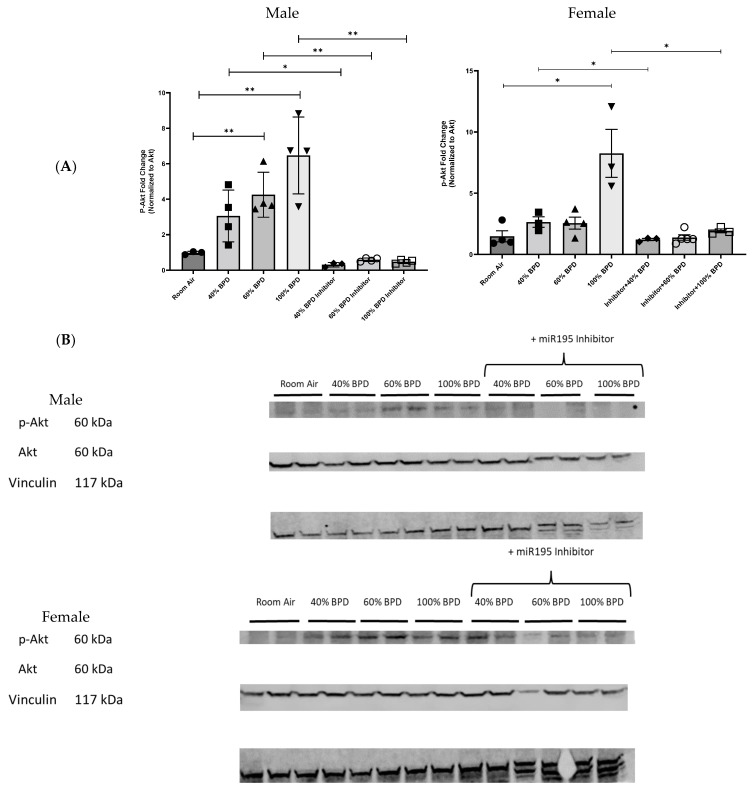
miR-195 inhibition decreased p-Akt expression in the lungs of NB male and female mice with 40%, 60%, and 100% O_2_ hyperoxia-induced experimental BPD. (**A**) Fold-change of relative protein expression. (**B**) Representative Western blot image showing decreased p-Akt expression in 40%, 60%, and 100% hyperoxia-induced BPD with miR-195 inhibition. Each well represents lung tissue from an individual mouse. n = 3–4. * *p* < 0.05, ** *p* < 0.01.

**miR-195 upregulates p-Akt in developing lungs upon exposure to varying concentrations of oxygen.** To further explore the mechanisms of miR-195 activity, we investigated a known downstream target protein of PHLPP2, the serine/threonine-specific protein kinase, Akt. To investigate the role of Akt in miR-195-mediated BPD, we analyzed relative p-Akt expression levels at 40%, 60%, and 100% O_2_-induced experimental BPD and after the administration of the miR-195 inhibitor. Consistent with the previously observed decrease in PHLPP2 in O_2_-induced BPD lung tissue, Akt was phosphorylated in response to hyperoxia. (Figure 3). Using the miR-195 inhibitor reduced p-Akt expression levels in 40%, 60%, and 100% BPD lung tissue in male mice. miR-195 inhibition in female mice significantly decreased p-Akt expression in the 40% O_2_ and 100% O_2_ hyperoxia-induced experimental BPD models, with a less substantial decrease in moderate BPD (Figure 3A,B). We examined the effect of administering miR-195 mimic and saw that Akt activation was increased in its presence (Supplemental Figure S3B). We also noted increased p53 and Bcl-2 expression in the 40% BPD model (and upon adding the miR-195 mimic), which was markedly decreased in the presence of the miR-195 inhibitor ( SupplementalFigure S3B).

As a surrogate marker for alveolar-capillary leak and/or lung injury, we also measured the BALF total protein concentrations. As noted in Supplemental Figure S4, the BALF protein concentration significantly increased with the increasing severity of the hyperoxia-exposed BPD models. In addition, the BALF total protein concentration was significantly increased in the 40% BPD model with miR-195 mimic compared to RA alone. Notably, the use of miR-195 inhibitor with 40% BPD resulted in significantly decreased BALF total protein levels compared to 40% BPD with miR-195 mimic as well as with 60% BPD alone. (Supplemental Figure S4). 

We then decided to look at another known target protein (Mitofusin 2) of miR-195 independent of the PHLPP2-Akt signaling cascade to establish a larger association of miR-195 expression with oxidative stress and BPD pathology [15].

**Figure 4:**
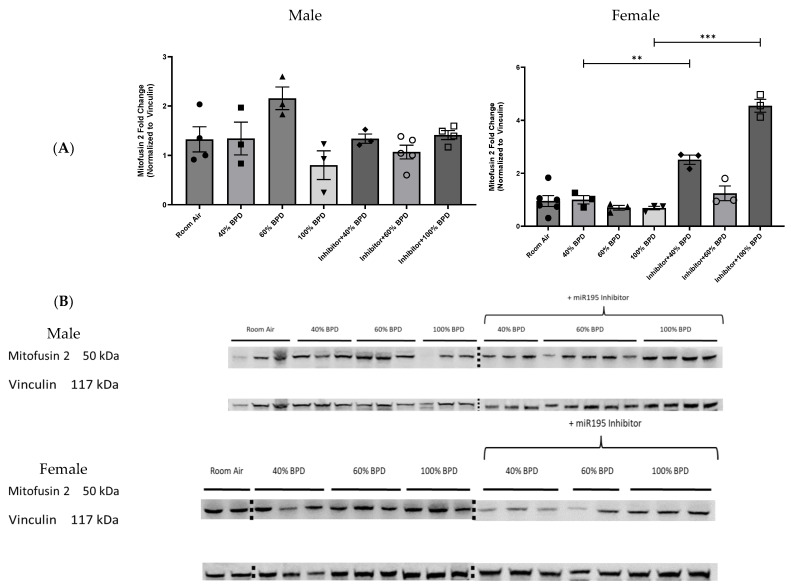
Impact of miR-195 inhibition on Mitofusin 2 expression in the lungs of NB male and female mice with 40%, 60%, and 100% O_2_ hyperoxia-induced experimental BPD. (**A**) Fold-change of relative protein expression. (**B**) Representative Western blot image showing Mitofusin 2 expression in 40%, 60%, and 100% hyperoxia-induced experimental BPD with miR-195 inhibition. Each well represents lung tissue from an individual mouse. n = 3–4. ** *p* < 0.01, *** *p* < 0.001.

**miR-195 downregulates Mitofusin 2 in developing lungs in the female BPD model upon exposure to varying concentrations of oxygen.** To investigate the role of Mitofusin 2 in hyperoxia-mediated BPD, we analyzed Mitofusin 2 expression levels at 40%, 60%, and 100% O_2_ hyperoxia-induced BPD as well as after the administration of the miR-195 inhibitor. Interestingly, we found no significant difference in Mitofusin 2 levels in the male BPD mouse model with the different levels of hyperoxia, with or without the miR-195 inhibitor (Figure 4A,B). In the female BPD mouse model, Mitofusin 2 levels were unchanged with increasing concentrations of O_2_ (increasing severity of BPD) (Figure 4A,B). Using the miR-195 inhibitor, however, increased Mitofusin 2 expression levels in 40% O_2_ (mild) and 100% O_2_ (severe) BPD lung tissue in female mice (Figure 4A,B). 

The next step in our investigation was to look at inflammatory markers that may be induced by the presence of miR-195 in neonatal BPD to demonstrate a possible causal association of miR-195 with the chronic inflammation that is characteristic of BPD [2].

**Figure 5:**
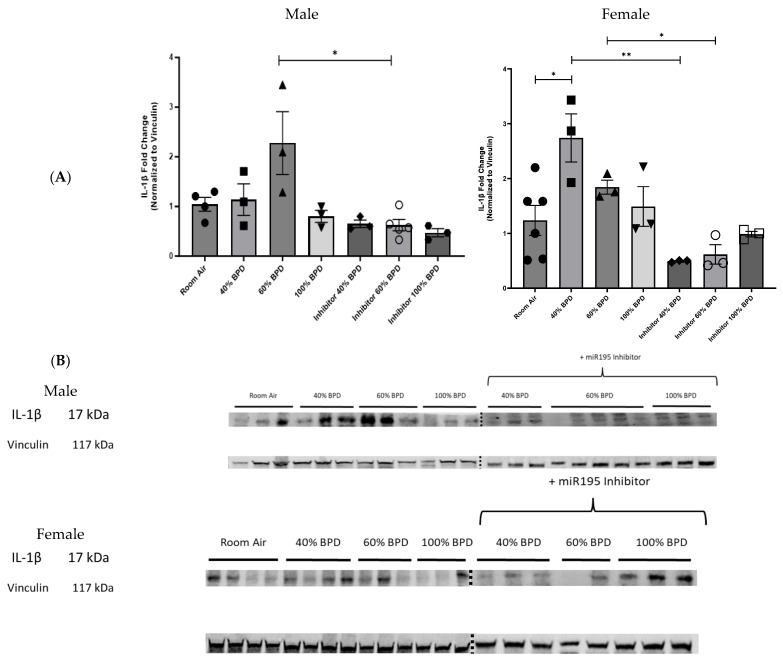
miR-195 inhibition decreased interleukin (IL)-1β expression in the lungs of NB male and female mice with hyperoxia-induced experimental BPD. (**A**). Fold-change of relative protein expression. (**B**). Representative Western blot image showing IL-1β expression in 40%, 60%, and 100% hyperoxia-induced BPD with miR-195 inhibition. Each well represents lung tissue from an individual mouse. Note: Vinculin blots for the males and females are the same as in Figure 4B. n = 3–4. * *p* < 0.05, ** *p* < 0.01.

**miR-195 upregulates IL-1β in developing lungs upon exposure to varying concentrations of oxygen.** To investigate the role of IL-1β in miR-195-mediated BPD, we analyzed relative IL-1β expression levels at 40%, 60%, and 100% O_2_ hyperoxia-induced experimental BPD and after the administration of the miR-195 inhibitor. IL-1β levels were increased proportionally to the amount of oxygen applied (Figure 5). Treatment with the miR-195 inhibitor, on the other hand, significantly decreased IL-1β expression levels in 60% O_2_ (moderate) BPD lung tissue in male as well as in 40% O_2_ (mild) and 60% O_2_ (moderate) BPD female mice. (Figure 5A,B).

**Figure 6:**
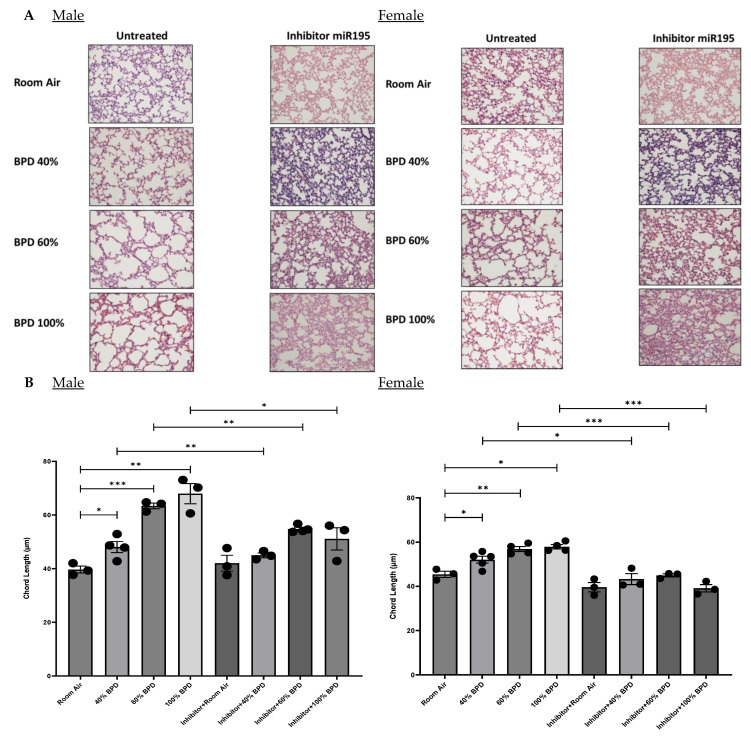

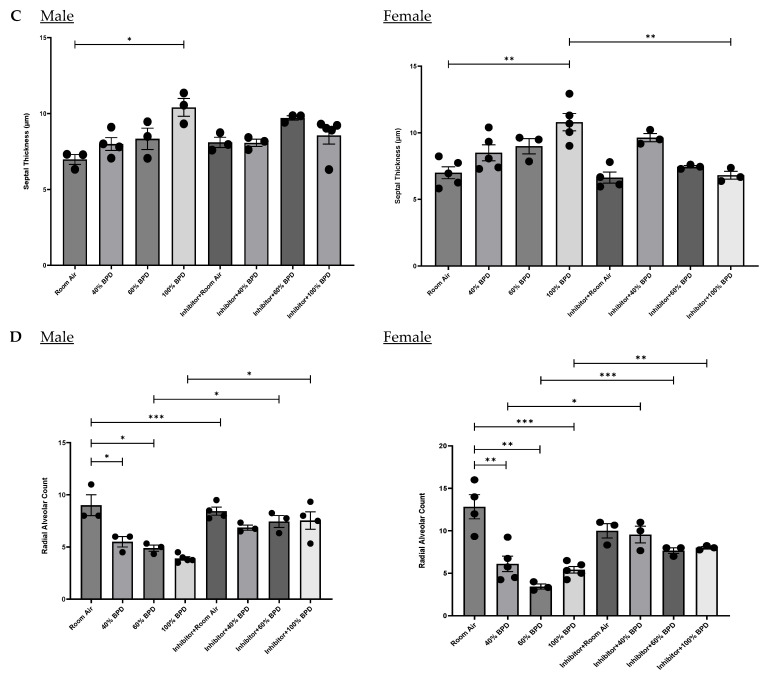
Morphological assessments of the developing mouse lung following O_2_-induced BPD. miR-195 inhibition improved BPD morphology in hyperoxia-induced experimental BPD. (**A**). H&E staining showing histology (shown at 20× magnification) of male and female mouse lungs for RA, BPD (40%, 60%, and 100% O_2_-induced), and BPD treated with miR-195 inhibitor specimens. In the BPD group, the alveoli are larger and simplified compared with RA controls, with recovery upon miR-195 inhibition. (**B**). Bottom panels show improvement in (**B**) chord length, (**C**) septal thickness, and (**D**) radial alveolar count after treatment with the miR-194 inhibitor. n= 3–4 mice. * *p* < 0.05, ** *p* < 0.01, *** *p*< 0.001.

**miR-195 inhibition improves hyperoxia-induced BPD morphology caused by varying concentrations of O_2_ exposure in NB mouse lungs.** Histologically, BPD is characterized by large and simplified alveoli. Figure 6 shows the lung architecture (Figure 6A), chord length (Figure 6B), septal thickness (Figure 6C), and the radial alveolar count (Figure 6D) for BPD in H&E-stained lung sections for both male and female mice pups. The increase in chord length and septal thickness was proportional to the concentration of O_2_ applied to induce BPD with the highest values for these parameters observed at 100% O_2_ (severe) BPD. After treatment with the miR-195 inhibitor, the chord length measurements were noted to be approaching RA control values in the 40%, 60%, and 100% O_2_ hyperoxia-induced lungs (Figure 6B). Septal thickness decreased with miR-195 inhibition in female mice towards RA control levels in 100% O_2_-induced BPD mice (Figure 6C). Radial alveolar counts showed a decrease in BPD and a significant recovery towards RA control values after treatment with the miR-195 inhibitor in male and female mice (Figure 6D).

**Clinical Relevance: Hyperoxia induces miR-195 expression in human TA.** A total of eight human TA samples were collected, five of which were from infants who eventually developed BPD (n = 5) and the rest from infants who did not develop BPD (n = 3). The no-BPD group were all males, the BPD group had one female, and the rest were males. The mean gestational age (GA) of infants with no BPD was 26.1 ± 0.2 weeks versus 25.9 ± 0.9 (*p* = 0.308) weeks in the BPD group, while the mean birth weight of infants with no BPD was 920.3 ± 17.6 g versus 591.3 ± 87.3 g (*p* = 0.002) in the BPD group.

**Figure 7:**
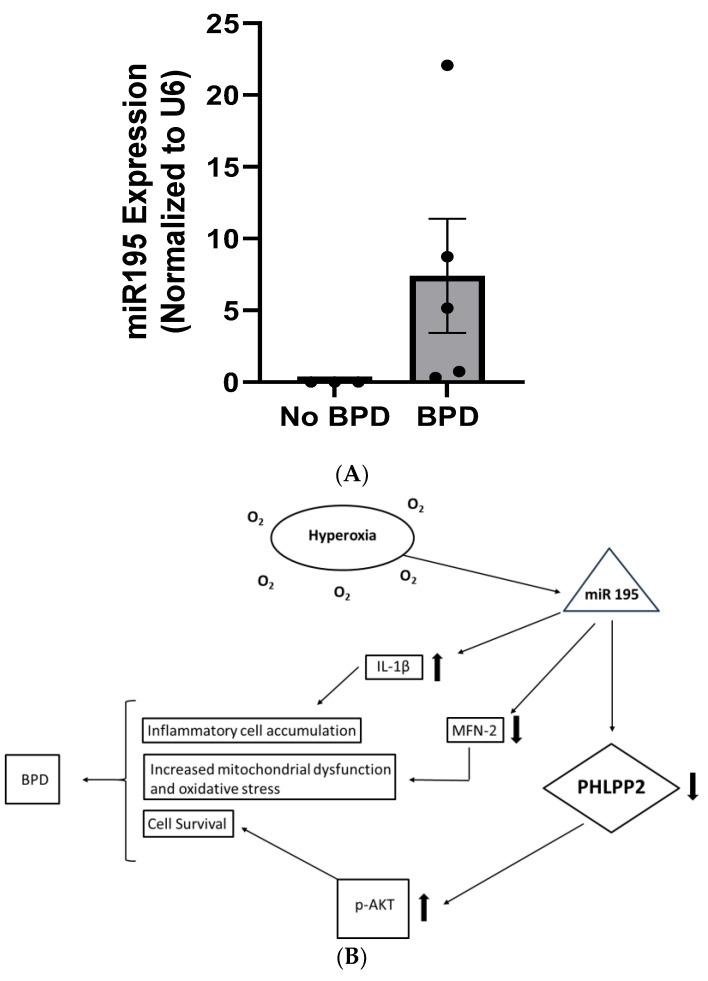
(**A**). Expression of miR-195 in tracheal aspirates of infants who developed bronchopulmonary dysplasia (BPD) compared to those who did not develop BPD. miR-195 expression was increased in the TAs of infants with BPD as compared to infants who did not develop BPD. No-BPD (n = 3), BPD (n = 5); Student’s one-tailed unpaired *t*-test: *p* = 0.068. (**B**). Proposed schematic representation of the role of miR-195 in hyperoxia-induced mild–moderate BPD. Hyperoxia exposure to the developing lung results in increased expression of miR-195, which causes decreased levels of PHLPP2 and increased levels of p-Akt. Constitutive activation of Akt contributes to increased antiapoptotic signaling, recruitment, and accumulation of inflammatory cells and tissue remodeling. Inflammatory cytokines such as interleukin (IL)-1β are upregulated whereas pro-survival proteins such as Mitofusin (MFN2) are downregulated. The combination of these factors may contribute, at least in part, to the compromised pulmonary phenotype characteristic of BPD.

We found that miR-195 showed a trend (*p* = 0.068) towards an increased expression in TA of infants with BPD as compared to infants who did not develop BPD (Figure 7A). This finding emphasized the translational significance of miR-195 in BPD in preterm human infants.

Figure 7B shows a proposed scheme for the role of miR-195 in the pathogenesis of BPD.

## 4. Discussion

miRs have been implicated in the regulation of hyperoxia-induced injury and cell death in the developing lung [10]. In the present study, we present four major novel findings that demonstrate the importance of miR-195 in hyperoxia-induced BPD. First, hyperoxia consistently induced miR-195 expression in mouse lung epithelial cells (TIIAECs) and in the whole-lung lysates of NB mice, at moderate degrees of hyperoxia as well as in human TAs of infants who later went on to develop BPD. Second, we validated PHLPP2 as a potential signaling intermediate responsive to miR-195 and showed that PHLPP2 expression was inversely correlated to that of miR-195 in BPD. Third, Akt phosphorylation increased inversely to PHLPP2 activation and proportionately to the amount of oxygen utilized to induce BPD in mouse lung tissue (40%, 60%, and 100% O_2_). Fourth, we demonstrated the feasibility and efficacy of in vivo miR-195 inhibition, which effectively induced PHLPP2 expression and improved BPD morphology. The potential beneficial effect of the miR-195 inhibitor (and conversely, the detrimental effect of the miR-195 mimic) is supported by the total BALF protein concentration measured (as a surrogate marker for lung injury and/or alveolar-capillary leak) in mouse lungs. These data are the first to suggest that miR-195 may be a potential therapeutic target in hyperoxia-induced BPD at moderate levels of hyperoxia that induces Akt phosphorylation by suppressing PHLPP2, with miR-195 inhibition improving BPD morphology in the developing mouse lung.

We were able to demonstrate miR-195 upregulation at moderate degrees of hyperoxia, with the greatest relative expression at 60% O_2_ exposure. Most hyperoxia-BPD animal models are based on O_2_ concentrations greater than 70% O_2_ [16]. By reducing the O_2_ concentration to 40–60% O_2_, we were, therefore, able to validate and employ conditions that were previously established by our research laboratory and elsewhere for creating the optimal, most appropriate animal model to evaluate the effectiveness of lung therapies targeted at ameliorating hyperoxia-induced BPD in mice [11,17]. Critically, our data on using 40–60% O_2_ has translational importance given the fact that these concentrations of hyperoxia exposure are clinically relevant in the current care of premature infants in the NICU.

Hyperoxic conditions in the developing lung are associated with the development of BPD and lead to a release of various reactive oxygen species, such as hydrogen peroxide (H_2_O_2_) and superoxide (O_2_^−^), which can trigger mitochondrial fragmentation by decreasing the expression of pro-fusion proteins as well as the release of pro-inflammatory cytokines [18,19,20]. Purohit et al. had previously demonstrated a functional association of miR-195 expression with mitochondrial dysfunction and oxidative stress [15,21]. Our experiments could confirm those observations with decreased expression levels of Mitofusin 2 in the female BPD model when miR-195 was not inhibited (Figure 4) as well as an increased expression for IL-1β in both male and female BPD models (Figure 5). We were intrigued by the lack of a linear relationship between the different concentrations of O_2_ and the expression of IL-1β. While some of the differences could be sex-specific (60% O_2_ showing the maximal IL-1β levels in males versus 40% having the higher levels of IL-1β in females), this could possibly be further compounded by the complexity of IL-1β regulation. IL-1β is regulated at the transcription, post-transcription, and post-translational levels [22]. In addition, extensive tissue oxidative damage at the higher concentrations of hyperoxia exposure as well as rapid protein turnover could cause a failure in IL-1β protein accumulation [22].

The adverse biochemical environment in hyperoxic lungs likely contributes to cellular and molecular damage as well as to genetic mutations within the lung, including DNA deterioration. Additionally, in a wide range of respiratory diseases such as BPD, cell signaling circuitry associated with alveolar development such as Notch signaling, is reported to be dysregulated, which can then prompt the remodeling of the extracellular matrix, prevent apoptosis, and cause the aberrant formation of lung tissue [23]. All the aforementioned factors likely corroborate or could potentially be an indirect result of miR-195 signaling, inducing the presented morphological features characteristic of BPD such as alveolar simplification, septal thickening, and decreased radial alveolar count (Figure 6).

In addition, we show evidence of increased p53 expression in our experimental model of BPD (and with the miR-195 mimic), as has been reported elsewhere for the animal models of BPD as well as in human lungs with BPD [24,25,26,27]. The Bcl-2 family of proteins has been shown to be either pro- or anti-antiapoptotic, depending upon the pathophysiological context [28]. In our experimental model of 40% O_2_ BPD, (and with the miR-195 mimic), we found an increased expression of Bcl-2, as has also been reported elsewhere in a newborn rat model exposed to hyperoxia [29]. The use of the miR-195 inhibitor, in the experimental model of 40% O_2_ BPD, on the other hand, leads to decreased expression of p53 and Bcl-2, suggesting an overall antiapoptotic signaling under the present experimental conditions.

Furthermore, the results suggest that PHLPP2 can act as a molecular signaling target of miR-195 and may regulate processes such as cell survival within the immature mouse lung tissue. Gu et al. had shown previously that miR-195 signals through PHLPP2 to deactivate Akt [14]. PHLPP2 itself is a well-established modulator of Akt signaling [30]. In agreement with previous studies, we could observe PHLPP2 as a miR-195-responsive mediator of Akt dephosphorylation. Our data suggest that miR-195 can reduce the PHLPP2 expression of NB mice with O_2_ hyperoxia-induced BPD, and lead to a subsequent activation of Akt.

Putatively, during the evolution of BPD, neonatal lungs are constantly exposed to high levels of O_2_ and airborne pathogens, which could mean that Akt activity is maintained for long periods of time. The persistence of Akt activation might then play an important part in the accumulation of macrophages, neutrophils, and T-lymphocytes in the airways, parenchyma, and pulmonary vasculature, prompting a perpetually active immune response that is reflected in the chronic lung inflammation in BPD in infants [31,32].

At the same time, evidence for Akt upregulation as a promoter of a dysregulated immune response needs to be balanced against studies that have shown that the expression of Akt in experimental BPD preserves alveolar formation and that inhibition of Akt disrupts normal lung development by preventing apoptosis in the developing lung epithelium [33,34].

Furthermore, our data support sex-specific differences both in signaling and morphological parameters within our BPD model. For example, p-Akt was significantly increased in 60% BPD in males but not in females (Figure 3). The expression of Mitofusin 2 in males did not change in hyperoxia-induced BPD, with or without miR-195 inhibition, whereas in females it was upregulated in response to miR-195 inhibition in 40% and 100% O_2_ hyperoxia-induced BPD. Septal thickness was significantly improved in females with severe BPD upon miR-195 inhibition. The differences presented here illustrate a larger complexity of this disease with respect to sex-specific coping mechanisms and will, therefore, have to inform future research on BPD [6].

## 5. Conclusions

In conclusion, we show that miR-195 may contribute to neonatal hyperoxia-induced murine BPD by influencing cell survival through the regulation of the antiapoptotic PHLPP2/Akt signaling scaffold. The inhibition of miR-195 led to increased PHLPP2 activation, decreased p-Akt expression, and improvement in BPD morphology, particularly at moderate levels of hyperoxia exposure and in male mouse pups. The pharmacodynamic inhibition of miR-195 could, therefore, hold translational therapeutic value in the treatment of BPD and other respiratory diseases that currently lack sufficient treatment options.

## Data Availability

The original contributions (Western Blots) presented in the study are included in the article/Supplementary Materials; further inquiries can be directed to the corresponding author.

## Supplementary Materials

The following supporting information can be downloaded at: https://www.mdpi.com/article/10.3390/biomedicines12061208/s1, Figure S1: Experimental BPD modeling. Figure S2: Expression of miR-195 in 40% hyperoxia exposed neonatal cell lines. Figure S3: Representative Western blot image showing PHLPP2, AKT, p-AKT, Bcl-2 and p53 expression in 40% hyperoxia-induced experimental BPD with miR-195 mimic and inhibitor. Figure S4: BALF protein concentration in NB mice exposed to hyperoxia with miR-195 mimic and inhibitor. Western Blots raw data (PDF).
