# Supplementary material for: Hyperoxia-Induced miR-195 Causes Bronchopulmonary Dysplasia in Neonatal Mice
Source: Biomedicines. 2024 May 29;12(6):1208. doi: 10.3390/biomedicines12061208 (PMC11201213; doi:10.3390/biomedicines12061208)
Supplement: Supplementary file 1 [file biomedicines-12-01208-s001.zip › biomedicines-2991401-supplementary.pdf]

biomedicines

MDPI

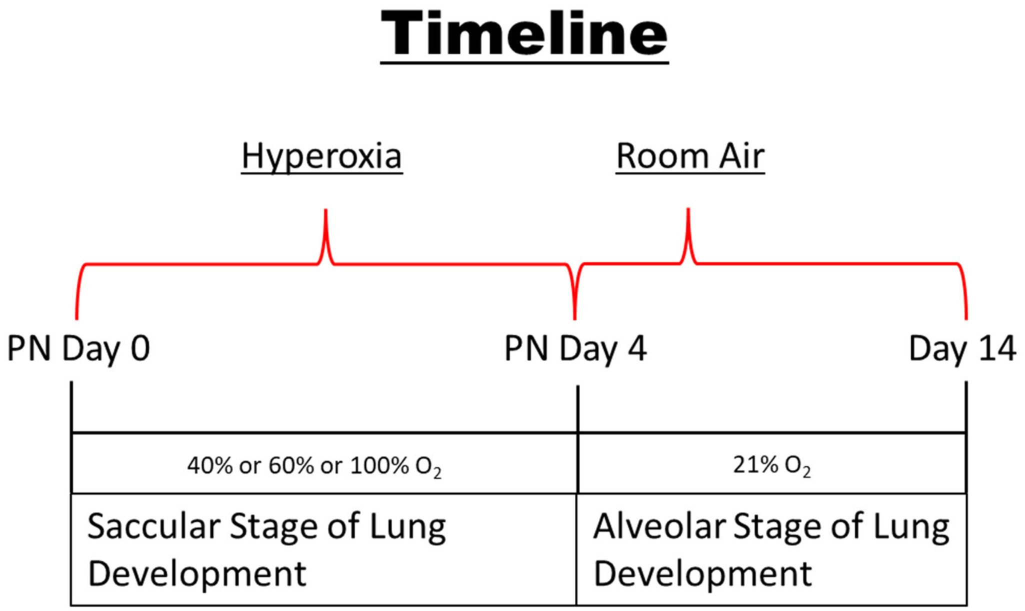

Figure S1. For hyperoxia experiments, NB mice were exposed to specific concentrations of oxygen along with their mothers for different time points. Hyperoxia (40% or 60%, or 100% O_2) exposure occurred from PN day 1 to PN day 4 which corresponds to the saccular stage of lung development in rodents. Mice were then allowed to recover in RA for 10 days (i.e. during the alveolar stage of lung development). Lungs were then harvested at PN day 14. NB: newborn; PN: postnatal.

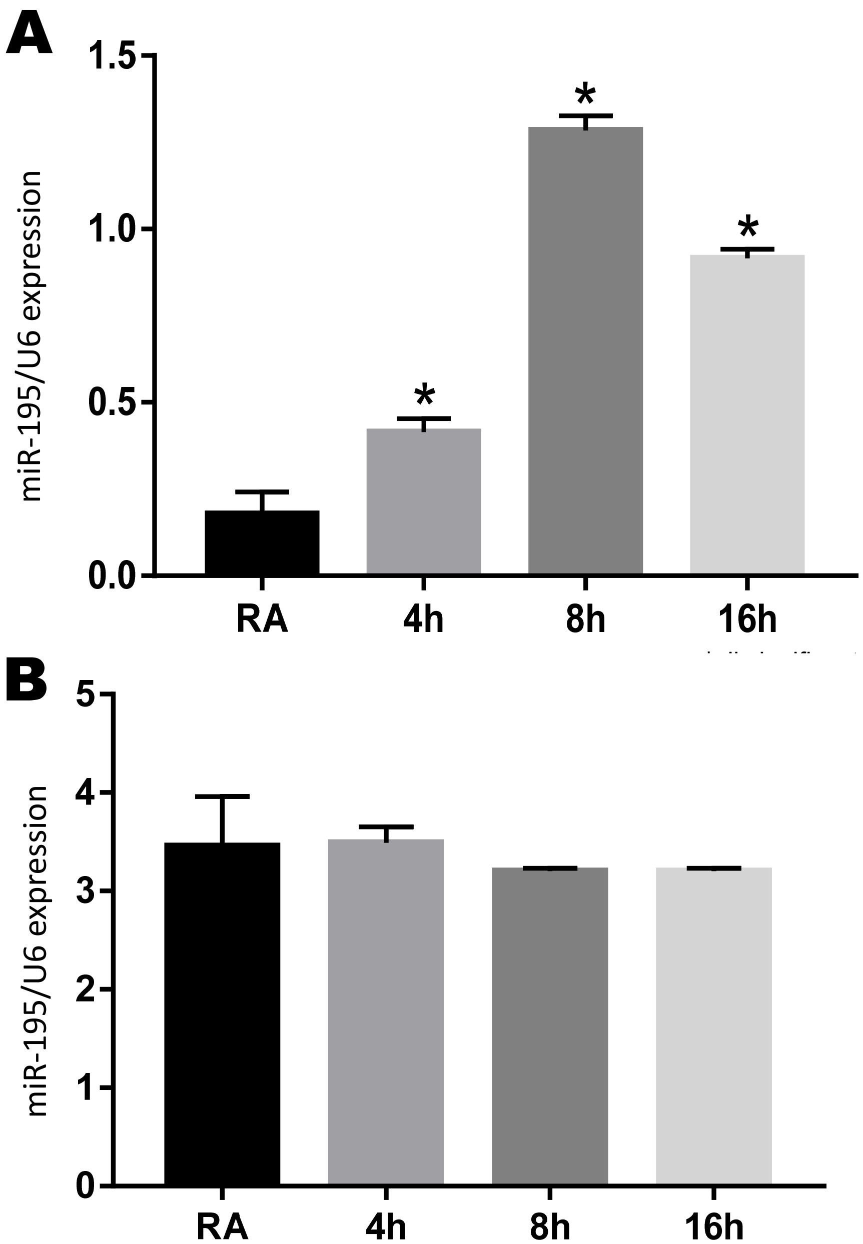

Figure S2. Expression of miR-195 in 40% hyperoxia exposed neonatal cell lines. **A.** Type II alveolar epithelial cells (TIIAECs) were exposed to 40% oxygen for different time points, harvested and RNA extracted. **B.** Neonatal lung endothelial cells were exposed to 40% for different time points, harvested and RNA extracted. miR-195 expression was normalized to RNU6. RA: room air. *p <0.05 compared to room air (RA) controls.

A

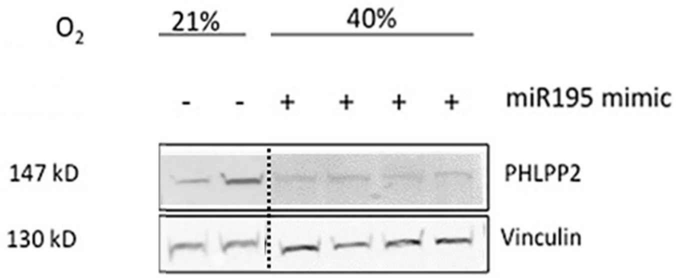

B

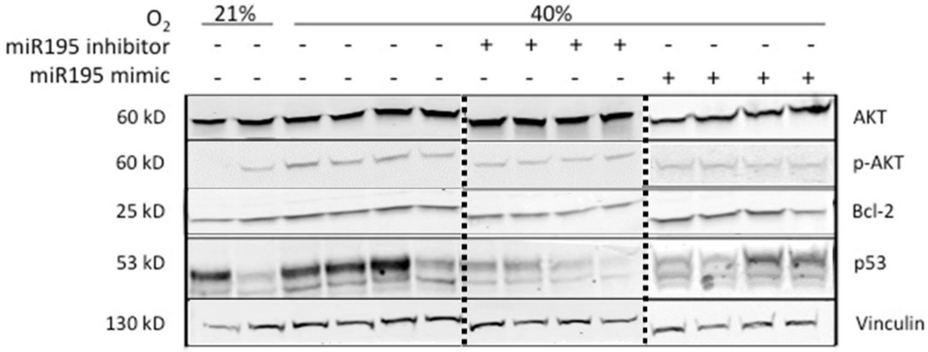

Figure S3. A. Administration of miR-195 mimic decreased PHLPP2 expression. Each well represents lung tissue from an individual mouse pup. **B.** miR-195 directly targets the PHLPP2-Akt-apoptosis pathway in the lungs of NB mice with 40% hyperoxia-induced experimental BPD. Western blot image showing increased p-Akt expression in 40% hyperoxia induced experimental BPD. Akt activation (p-Akt) is decreased in mild hyperoxia-induced BPD when miR-195 is inhibited, and it is increased with treatment with the miR-195 mimic. Bcl-2 and p53 are increased in 40% hyperoxia, decreased following miR-195 inhibition, and increased with treatment with the miR-195 mimic. Note: Vinculin blot for the miR-195 mimic with 40% O_2 is the same as in **A**. n=3-4. NB: newborn; BPD: bronchopulmonary dysplasia.

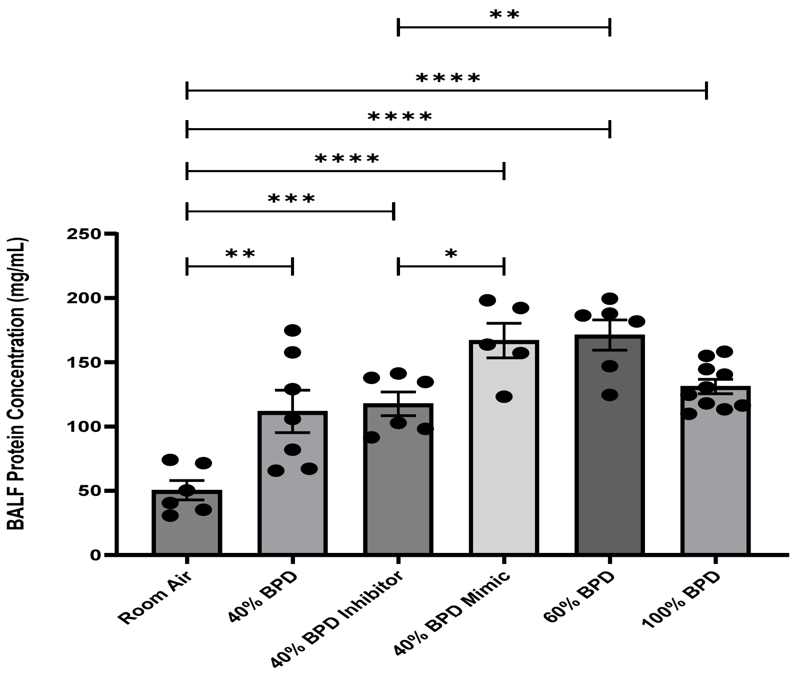

Figure S4. Hyperoxia increased BALF protein concentration in NB mice. BALF was collected from airways of NB mice within the 40%, 60% and 100% hyperoxia-induced BPD model groups. Protein concentration was significantly increased above the control in all levels of hyperoxia. BALF protein concentration increased after treatment with the miR-195 mimic and decreased with the use of miR-195 inhibitor. At least 5 samples were collected in each group. *$p < 0.05$, **$p < 0.01$, ***$p < 0.001$, **** $p \le 0.0001$. BALF: bronchoalveolar lavage fluid; NB: newborn; BPD: bronchopulmonary dysplasia.
