# Supplementary material for: Hyperoxia-Induced miR-195 Causes Bronchopulmonary Dysplasia in Neonatal Mice
Source: Biomedicines. 2024 May 29;12(6):1208. doi: 10.3390/biomedicines12061208 (PMC11201213; doi:10.3390/biomedicines12061208)

Western Blot Raw Data

Fig.2B

PHLPP2

Male - BPD

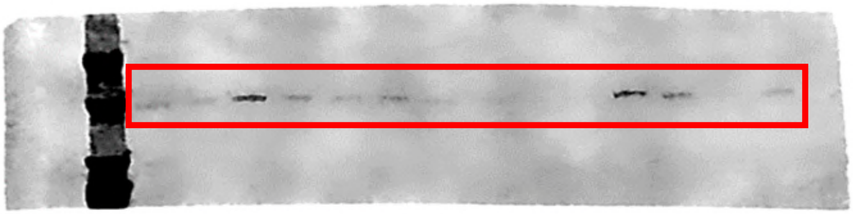

Vinculin

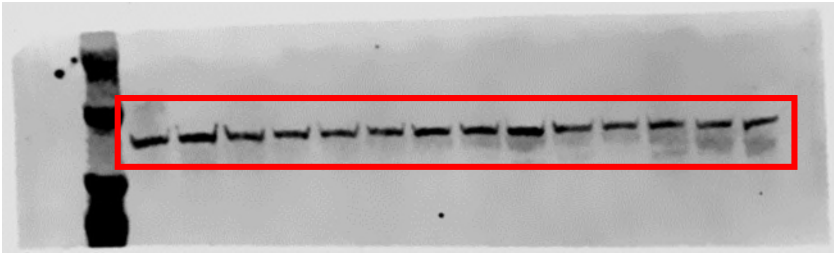

Female - BPD

PHLPP2

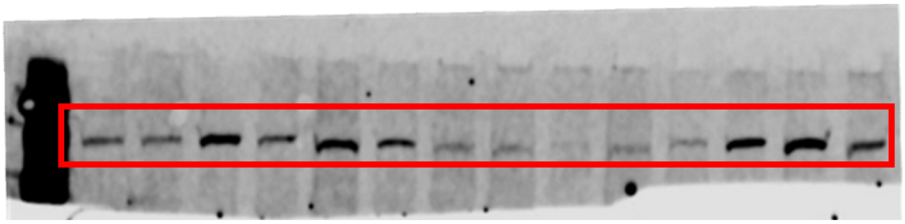

Vinculin

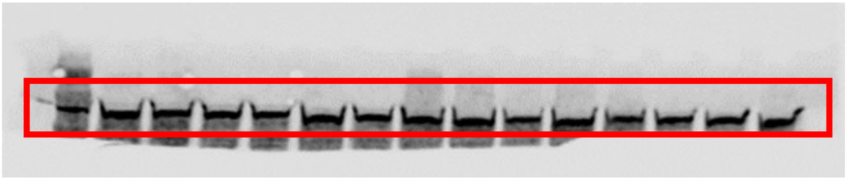

Fig.2B

Male - miR195 inhibitor

PHLPP2

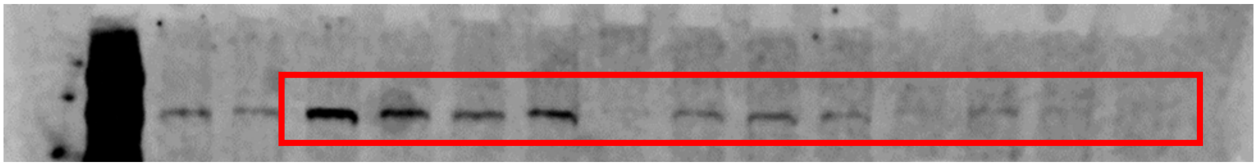

Vinculin

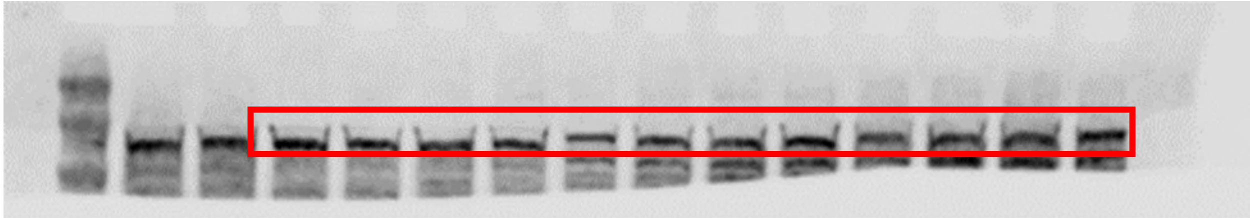

Female - miR195 inhibitor

PHLPP2

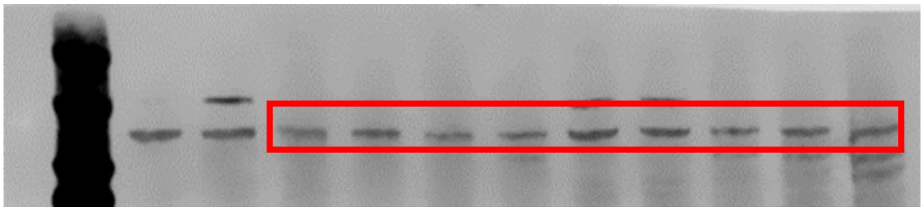

Vinculin

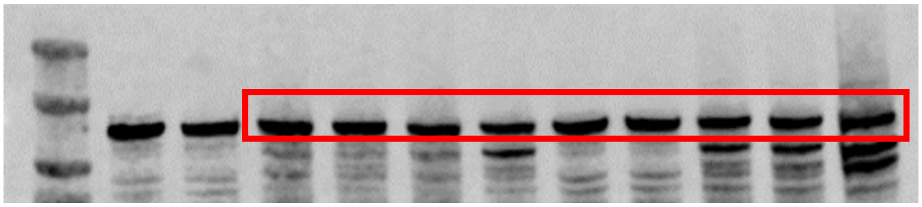

Fig.3B

Male – BPD and miR195 Inhibitor

p-Akt

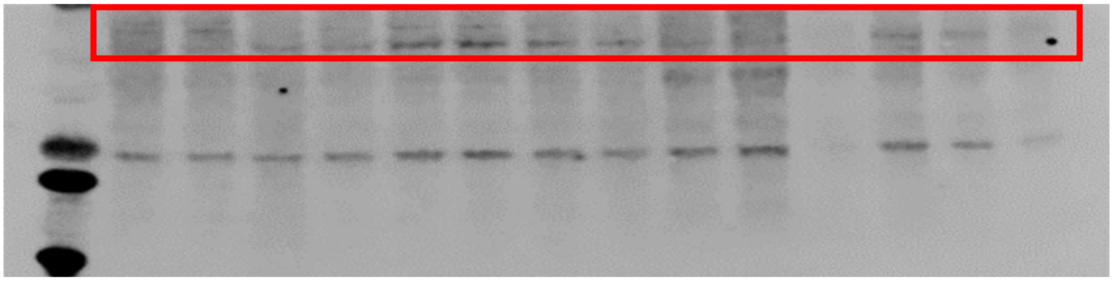

Vinculin

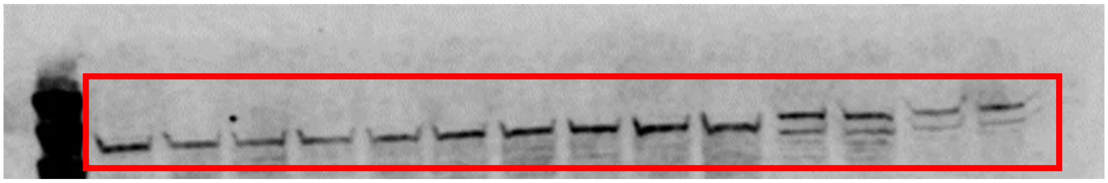

Akt

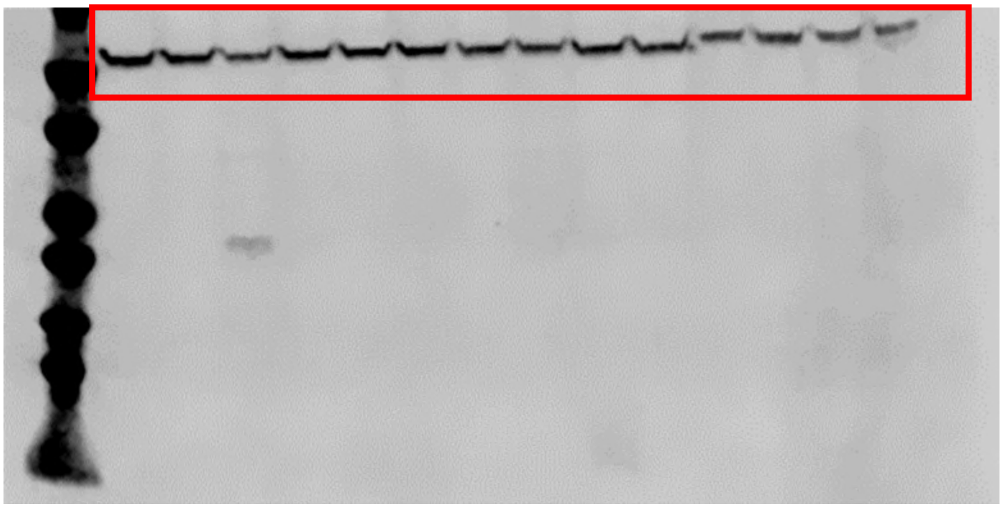

Fig.3B

Female - BPD and miR195 Inhibitor

p-Akt

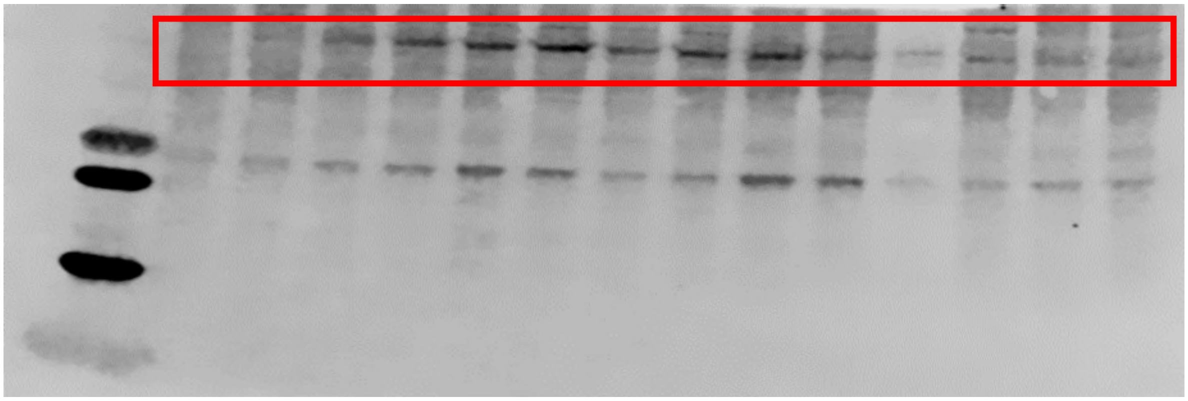

Vinculin

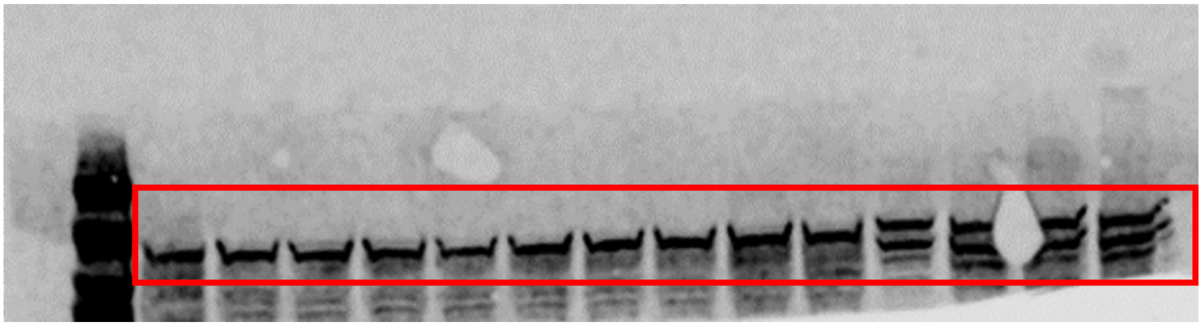

Akt

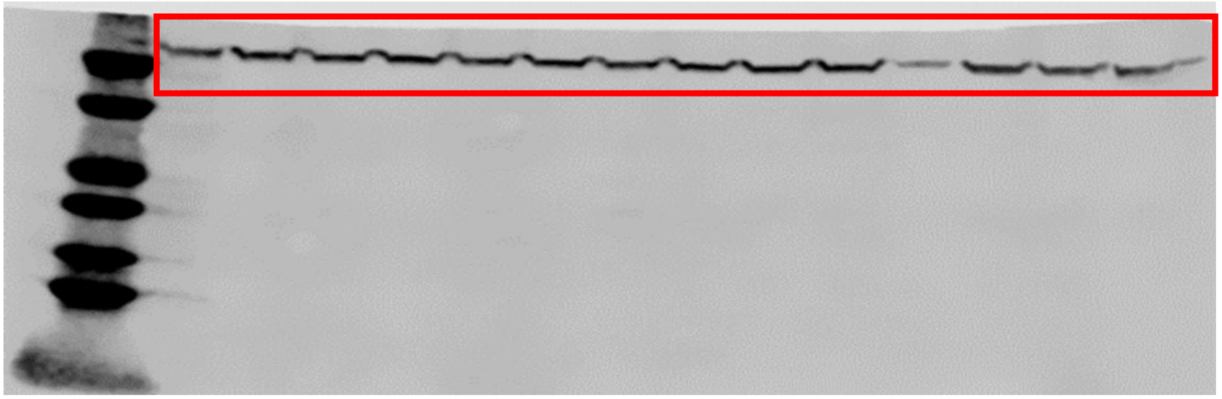

Fig.4B

Male - BPD

Mitofusin 2

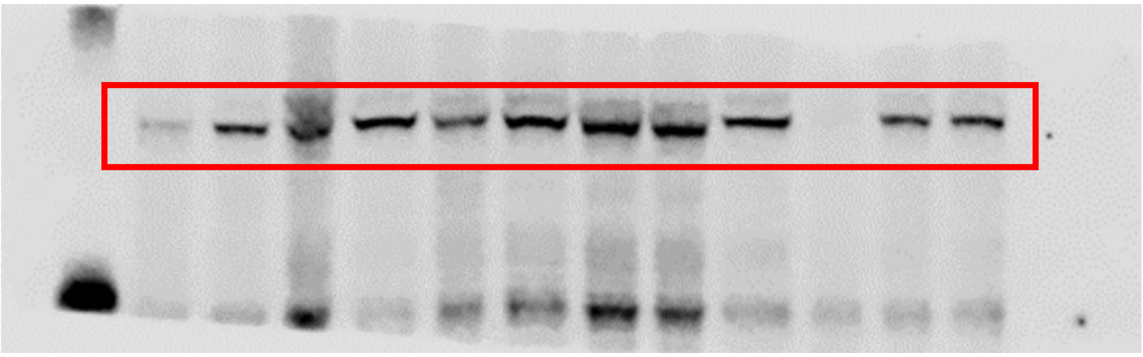

Vinculin

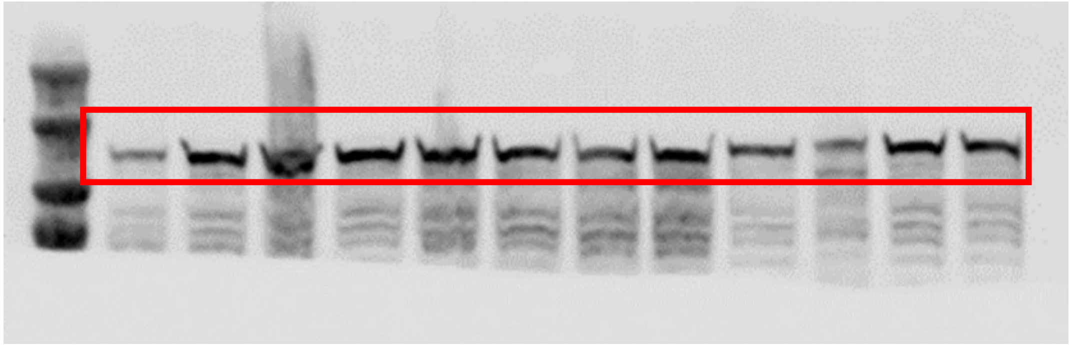

Fig.4B

Female - BPD

Mitofusin 2

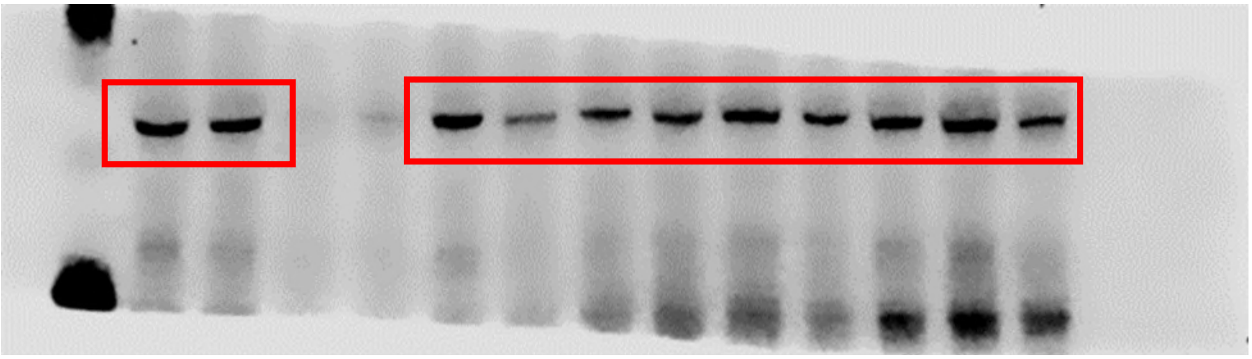

Vinculin

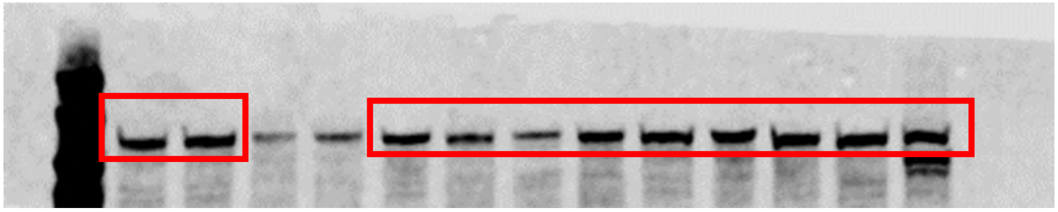

Fig.4B

Male - miR195 Inhibitor

Mitofusin 2

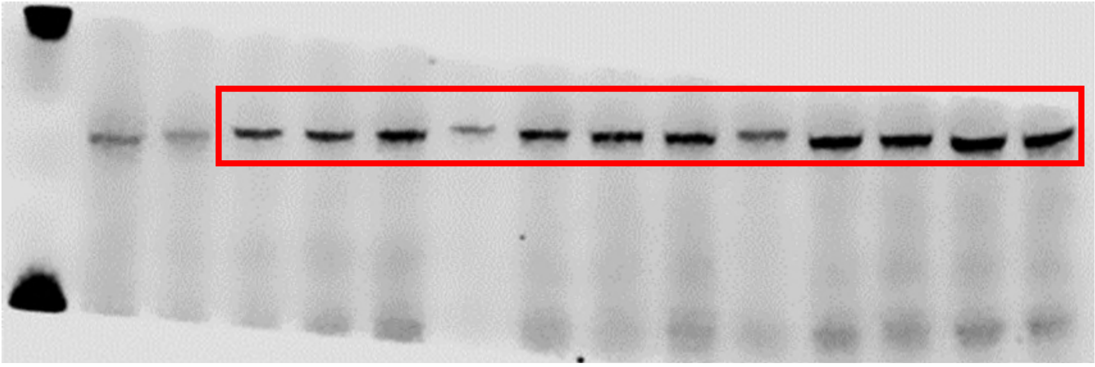

Vinculin

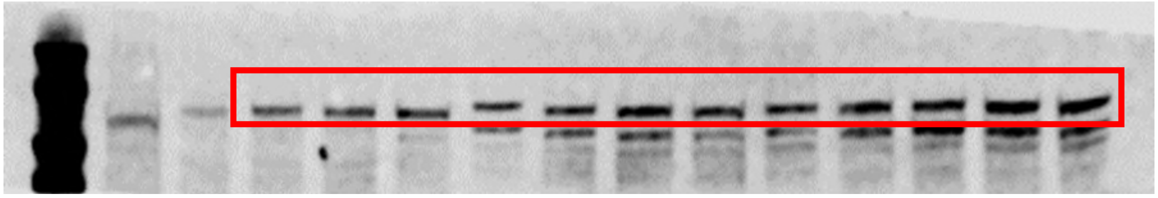

Fig.4B

Female - miR195 Inhibitor

Mitofusin 2

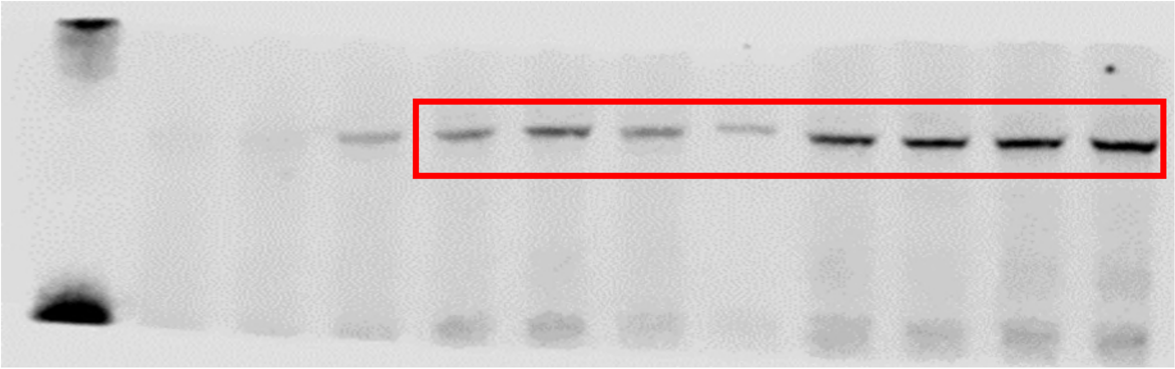

Vinculin

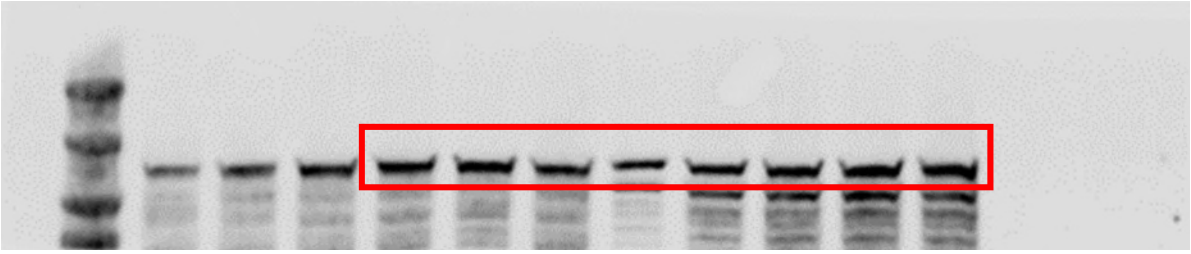

Fig.5B

Male - BPD

IL-1β

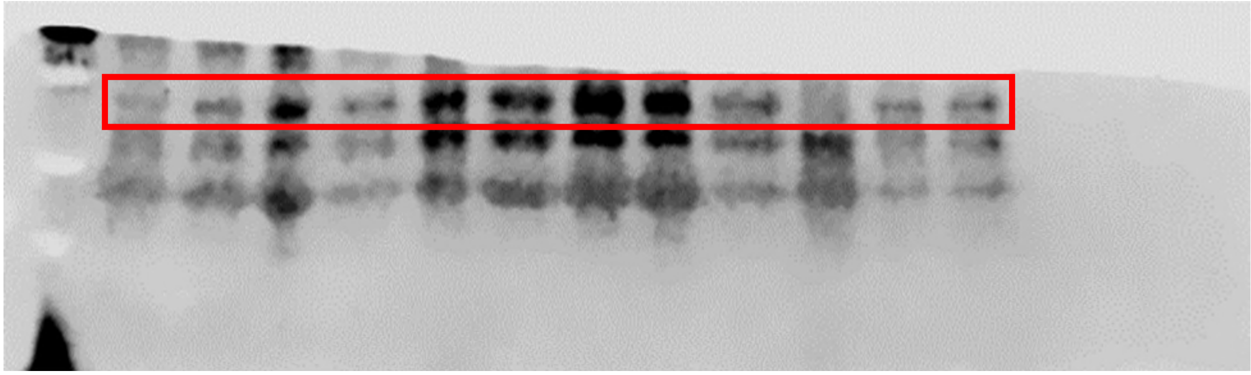

Vinculin

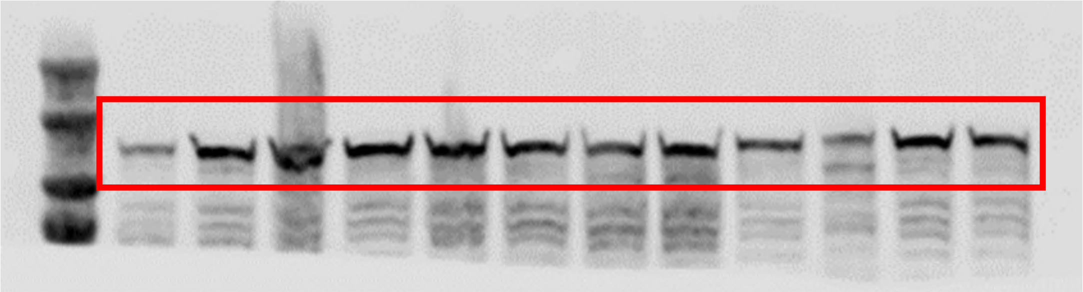

Fig.5B

Female - BPD

IL-1β

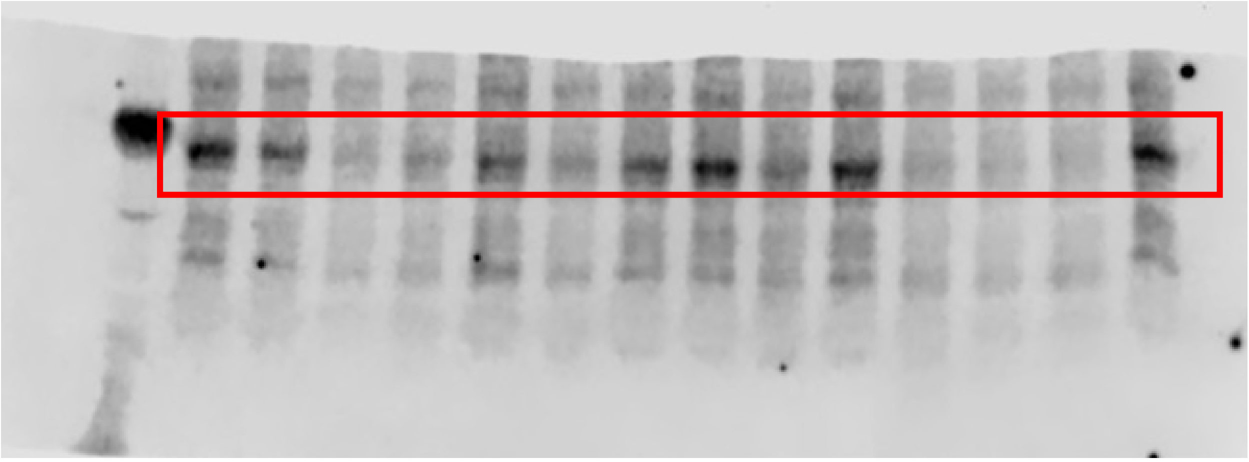

Vinculin

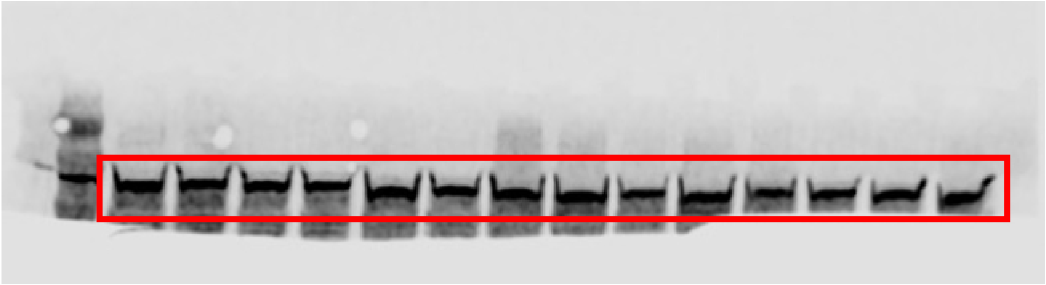

Fig.5B

Male - miR195 Inhibitor

IL-1β

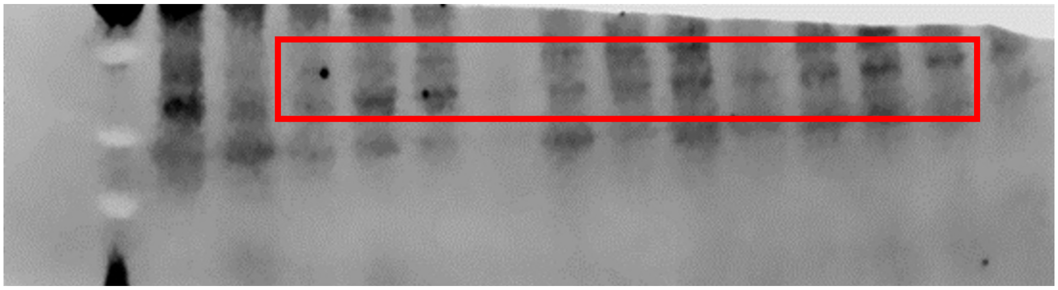

Vinculin

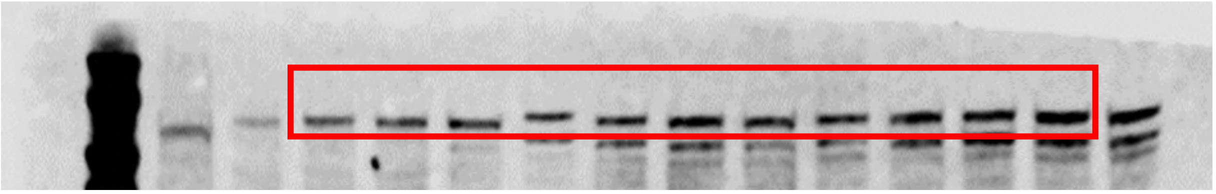

Fig.5B

Female - miR195 Inhibitor

IL-1β

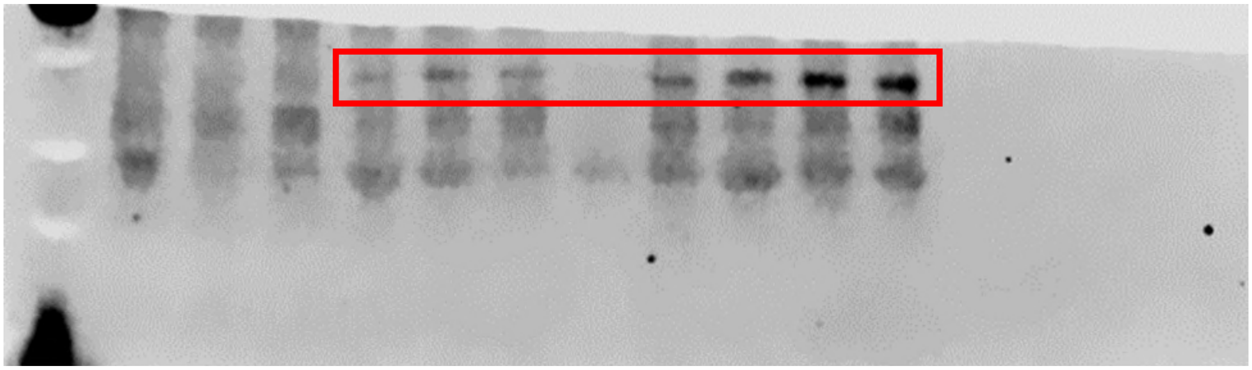

Vinculin

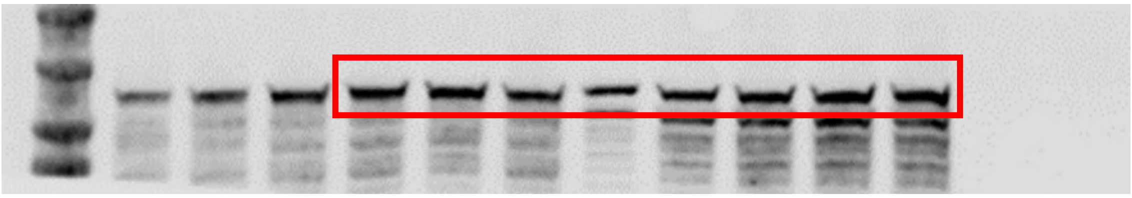

Supplemental Fig.3A

PHLPP2 control lanes 1-2

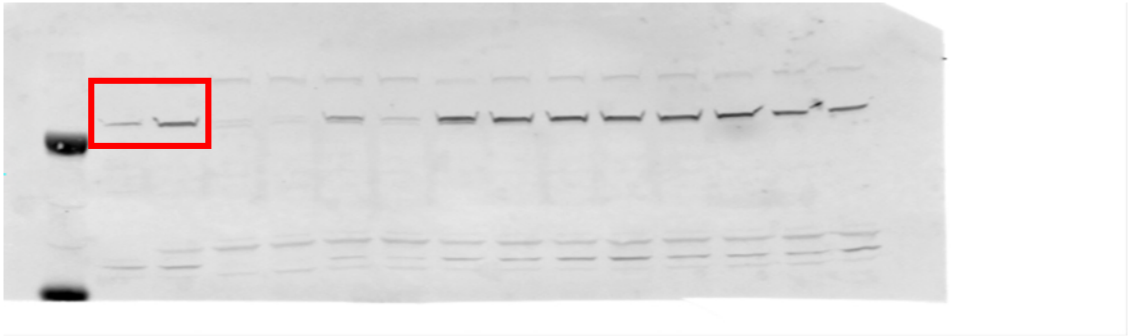

PHLPP2 Vinculin control lanes 1-2

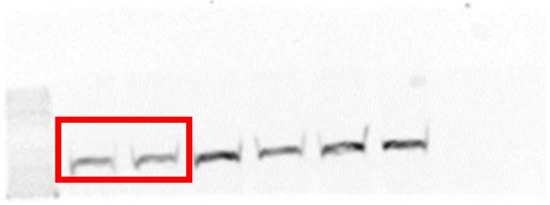

PHLPP2 mimic lanes 3-6

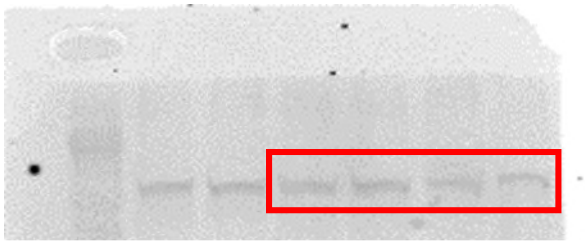

Vinculin mimic lanes 3-6

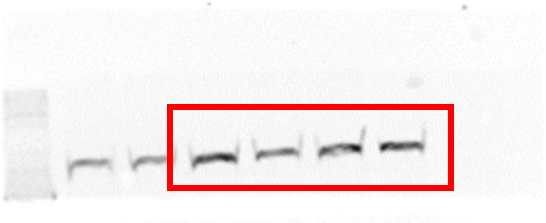

Supplemental Fig.3B

p53 control for inhibitor and mimic lanes 1-6

p53 inhibitor lanes 11-14

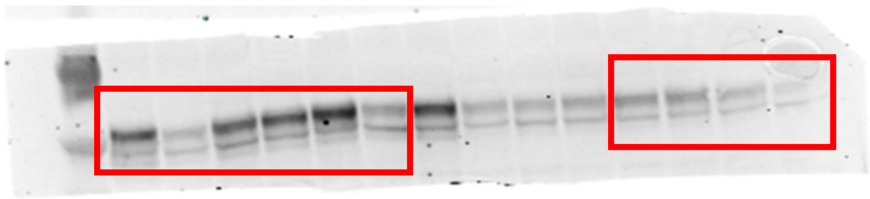

P53 mimic lanes 5-8

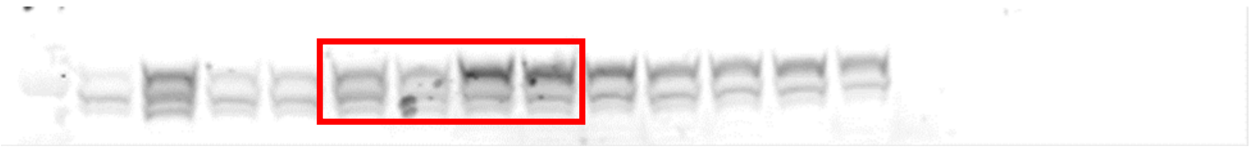

Supplemental Fig.3B

Bcl-2 control for inhibitor and mimic lanes 1-6

Bcl-2 inhibitor lanes 11-14

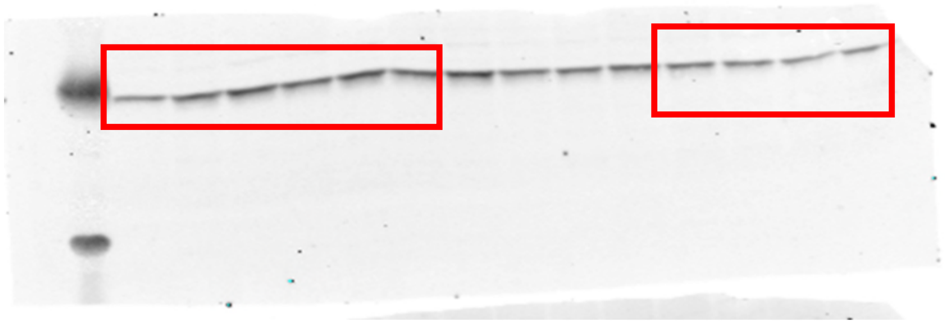

BCL-2 mimic lanes 3-6

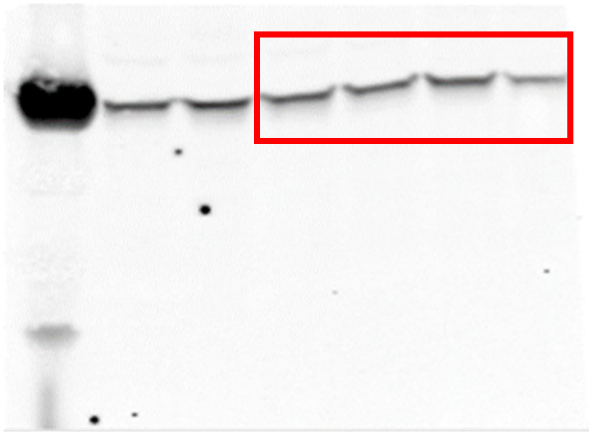

Supplemental Fig.3B

AKT control lanes1-6

AKT inhibitor lanes 11-14

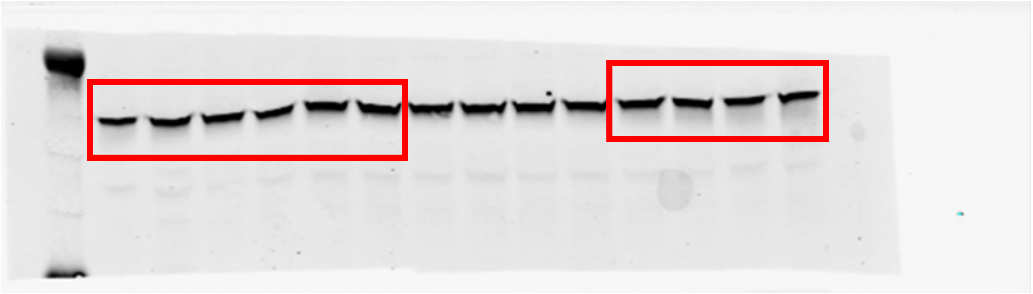

AKT mimic lanes 2-5

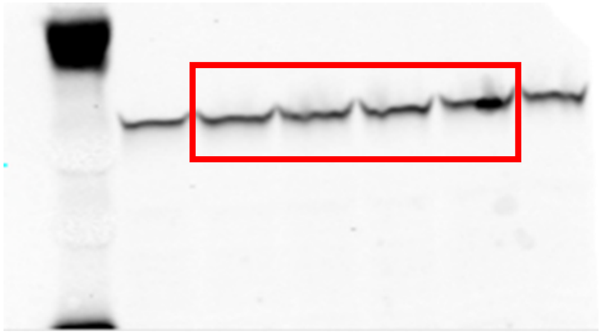

Supplemental Fig.3B

p-AKT control lanes 1-6

p-AKT inhibitor lanes 11-14

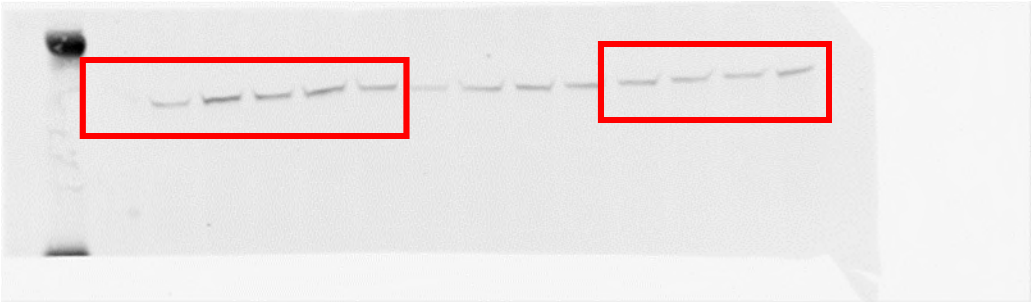

p-Akt mimic lanes 2-5

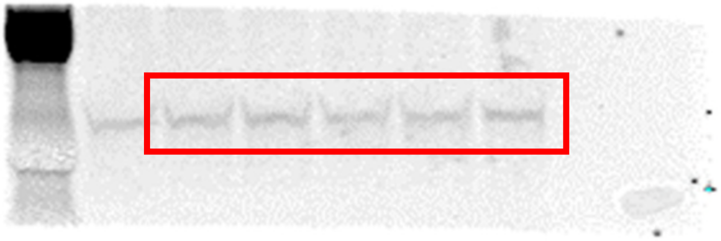

Supplemental Fig.3B

Vinculin control lanes 1-6

Vinculin inhibitor lanes 10-13

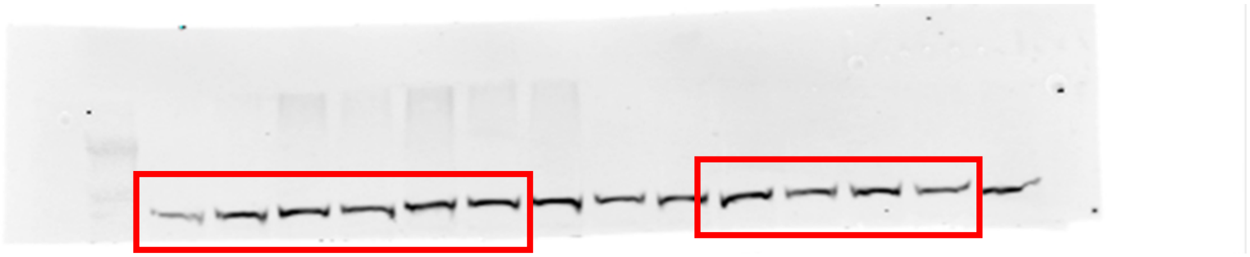

Vinculin mimic lanes 3-6

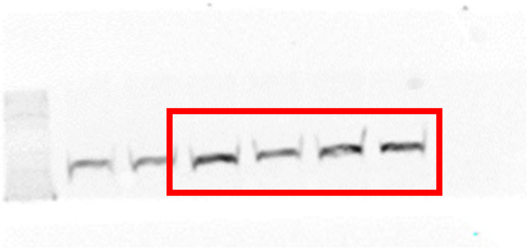

Supplement: Supplementary file 1 [file biomedicines-12-01208-s001.zip › WesternBlot_RawData_miR195REVISION2_FINAL_May212024.pdf]
